# The GM-JMNS-CPHD Filtering Algorithm for Nonlinear Systems Based on a Generalized Covariance Intersection

**DOI:** 10.3390/s24051508

**Published:** 2024-02-26

**Authors:** Zhixuan Xu, Yu Wei, Xiaobao Qin, Pengfei Guo

**Affiliations:** 1 School of Mathematics and Statistics, Hainan University, Haikou 570000, China; nick17681294553@163.com; 2 School of Information and Communication Engineering, Hainan University, Haikou 570000, China; 19806235003@163.com (Y.W.); 18788621543@163.com (X.Q.)

**Keywords:** generalized inverse covariance intersection, jumping Markov, GM-CPHD, nonlinear motion tracking

## Abstract

Some fusion criteria in multisensor and multitarget motion tracking cannot be directly applied to nonlinear motion models, as the fusion accuracy applied in nonlinear systems is relatively low. In response to the above issue, this study proposes a distributed Gaussian mixture cardinality jumping Markov-cardinalized probability hypothesis density (GM-JMNS-CPHD) filter based on a generalized inverse covariance intersection. The state estimation of the JMNS-CPHD filter combines the state evaluation of traditional CPHD filters with the state estimation of jump Markov systems, estimating the target state of multiple motion models without knowing the current motion models. The performances of the generalized covariance intersection (GCI)GCI-GM-JMNS-CPHD and generalized inverse covariance intersection (GICI)GICI-GM-JMNS-CPHD methods are evaluated via simulation results. The simulation results show that, compared with algorithms such as Sensor1, Sensor2, GCI-GM-CPHD, and GICI-GM-CPHD, this algorithm has smaller optimal subpattern assignment (OSPA) errors and a higher fusion accuracy.

## 1. Introduction

Random finite sets can solve the complex relationships of data in multitarget tracking. The commonly used filtering methods for random finite sets include probability hypothesis density (PHD) [1,2,3,4], penalized probability hypothesis density (CPHD) [5,6,7,8], generalized labeled multi-Bernoulli (GLMB) [9,10,11,12], and CPHD optimization algorithms, such as those used by Xu, W. (2023) [13], who proposed the Gaussian mixture (GM) implementation of HMB-CPHD filters in their research. Kim, S. Y. (2022) [14] proposed a sequential Monte Carlo-based cardinal probability hypothesis density (SMC-CPHD) filter in their study, and Li Y. (2022) [15] proposed a variational Bayesian expectation maximization method in their research and proposed a simulated Student t-distribution replacement PHD filtering method with multiple Gaussian mixture terms to achieve multiobjective tracking. In the process of multitarget tracking, there are often multiple sensors, and the results of multisensor fusion tracking are more time consistent, accurate, and reliable than those of a single sensor. Commonly used fusion strategies include the generalized covariance intersection (GICI), sequential inverse covariance intersection (SICI), parallel inverse covariance intersection (PICI), etc.; Wang, L. (2023) [16,17] proposed a parallel inverse covariance intersection Gaussian mixture cardinality probability hypothesis density (PICI-GM-CPHD) fusion strategy in their research, which utilizes the generalization ability of the PICI-GM-CPHD algorithm to effectively reduce the nonlinear complexity of the system. Liu, Y. (2021) [18] proposed a batch processing inverse covariance intersection BICI method in their research to achieve multisensor fusion localization; Qi, W. (2020) [19] validated the effectiveness of batch covariance intersection (BCI) fusion and fast sequential covariance intersection (SCI) fusion in their study; Jin, Y. (2020) [20] proposed a generalized covariance intersection (GCI) fusion method for random finite GICI sets in their research, which can effectively avoid label inconsistency sensitivity issues and target identity information loss; and Ajgl, J. (2022) [21] combined covariance intersection fusion with the upper bound of the joint mean square error matrix in their study. The problem of target tracking is actually the problem of tracking and filtering the target state, which requires accurately estimating the target state based on the target measurement data obtained by the sensor. Nonlinear filtering is a very important aspect of target tracking. In practical applications, almost all control systems are nonlinear, and linearity is an approximate description of nonlinearity to a certain extent [22]. Therefore, studying nonlinear filtering algorithms to reliably and accurately track targets is the main purpose of designing target tracking systems. The above fusion strategies are mainly aimed at achieving the fusion tracking of linear moving targets, and there is little research on fusion strategies for nonlinear systems.

Scholars have conducted in-depth research on the nonlinear motion tracking of multiple moving targets, such as García Fernándezngel (2020) [23], who proposed filters suitable for nonlinear motion tracking; Li, G. (2022) [24], who proposed a D-JDTC Bernoulli filter that can perform nonlinear motion tracking; and Vo, B. N. (2006) [25] et al., who proposed the JM-CPHD filter, which can be seen as extending the state integration in traditional CPHD filters to the double integration of mode and state. Yang, W. (2022) [26] and other scholars have used the Jump Markov system model to achieve filtering and tracking in their research. However, the above research mainly proposes filters for the nonlinear motion tracking of multiple moving targets without considering the fusion tracking between multiple sensors. Based on this, this study proposes a distributed Gaussian mixture cardinality jumping Markov CPHD filter based on a generalized inverse covariance intersection, which addresses the issues of the fusion criteria not being applicable to nonlinear motion models and the low fusion accuracy in multisensor and multiobjective motion tracking, achieving multisensor, multiobjective, and nonlinear motion tracking.

The target tracking problem is actually the problem of tracking and filtering of the target state, which is required to accurately estimate the target state based on the target measurement data obtained by the sensor [27,28]. Nonlinear filtering is a very important aspect of target tracking. In practical applications, almost all control systems are nonlinear, and linearity is an approximate description of nonlinearity to a certain extent. The above fusion strategies are mainly aimed at achieving the fusion tracking of linear moving targets, and there is relatively little research on fusion strategies for nonlinear systems. Based on this, this study proposes a distributed Gaussian mixture cardinality jumping Markov CPHD filter based on a generalized inverse covariance intersection, with its main contributions being implementing a state estimation of the JMNSCPHD filter by combining the state estimation of traditional CPHD filters with the state estimation of jump Markov systems; comparing multiple fusion strategies and exploring the fusion strategies applicable to GM-JMNS-CPHD in nonlinear systems; and verifying the effectiveness and robustness of the proposed algorithm through planned experiments.

## 2. Research Background

### 2.1. GM-CPHD Filter

The CPHD filter’s definition is based on a multiobject distribution and it encompasses processes that are independent and identically distributed. If we assume that the cardinality distribution p(n) of the point process is $\left|X\right|=n$, then the functional probability generation (PGFL) G[h] and the probability assumption density D(x) of the CPHD can be stated as

(1) $$f(X)≜n!\;\cdot \;p(n)\cdot f(x_{1})\cdots f(x_{n})$$

(2) $$G[h]=\sum_{n=0}^{\infty }p(n){\left(\int h(u)\cdot f(u)du\right)}^{n}$$

(3) $$v(x)≜\frac{\delta }{\delta x}{G[h]}_{\left|h=1\right.}=f(x)\sum_{n=1}^{\infty }n\cdot p(n)$$

1. Prediction of CPHD

(4) $$p_{k,t\left|t-1\right.}(n)=\sum_{j=0}^{n}p_{b}(n-j)\sum_{h=j}^{\infty }\left(\begin{array}{c} h\\ j \end{array}\right)p_{k,t}^{j}{\left(1-p_{k,t}\right)}^{h-j}p_{t-1\left|t-1\right.}(h)$$

(5) $$v_{k\left|k-1\right.}(x)=\int_{X}p_{s}(x^{\prime })p_{t}(x\left|x^{\prime }\right.)v_{k\left|k-1\right.}(x^{\prime })dx^{\prime }+v_{b}(x)$$

(6) $${\hat{N}}_{k\left|k-1\right.}={\hat{N}}_{a,k}+{\hat{N}}_{b,k}$$

where $p_{s}(x^{\prime })$ is a known objective transition function with a previous state and $p_{t}(x\left|x^{\prime }\right.)$ is the survival probability of a target with a previous state. ${\hat{N}}_{a,k}$ is the expected number of new goals, and ${\hat{N}}_{b,k}$ is the expected number of goals that survived from the time step k−1.

2. Updates to the CPHD

(7) $$p_{k,t\left|t\right.}(n)=\frac{L_{t}^{0}(d_{k,t\left|t-1\right.}(\cdot ),y_{t},n)p_{k,t\left|t-1\right.}(n)}{\sum_{i=0}^{\infty }L_{t}^{0}(d_{k,t\left|t-1\right.}(\cdot ),y_{t},i)p_{k,t\left|t-1\right.}(i)}$$

(8) $$d_{k,t\left|t\right.}(x)=L_{yt}(x)d_{k,t\left|t-1\right.}(x)$$

where $L_{yt}(x)$ is the generalized likelihood function.

The GM-CPHD tracker utilizes an estimated birth intensity and cardinality distribution to achieve intensity filtering for target tracking [29].

**Assumption 1.**  *Each moving target follows a linear Gaussian dynamic model.*

**Assumption 2.**  *The survival probability and detection probability of a moving target are not related to the motion state of the target.*

**Assumption 3.**  *The strength of the new target’s random set has a Gaussian mixture form.*

The recursive form of the GM-CPHD can be given as follows:

(1) Prediction of the GM-CPHD Filter

The Gaussian distribution form of $v_{k-1\left|k\right.(x)}$ is

(9) $$v_{k-1\left|k\right.}(x)=p_{s,k}\sum_{j=1}^{J_{k-1}}\omega_{k-1\left|k\right.}^{j}N(x;m_{k-1\left|k\right.}^{j},P_{k-1\left|k\right.}^{j})$$

where $\omega_{k}^{j}$ is the weight of the mixed newborn intensity, $m_{k}^{j}$ is the average of the mixed newborn intensity, and $P_{k}^{j}$ is the covariance of the mixed newborn intensity.

(10) $$m_{s,k\left|k-1\right.}^{j}=F_{k-1}m_{k-1}^{j}$$

(11) $$P_{s,k\left|k-1\right.}^{\left(j\right)}=Q_{k-1}+F_{k-1}P_{k-1}^{\left(j\right)}F_{k-1}^{Τ}$$

where $Q_{k-1}$ is the covariance matrix of the process noise; $F_{k-1}$ is the state transition matrix; and the Gaussian probability density function with covariance is P.

Assuming that the posterior intensity at time k−1 and the predicted time intensity are both Gaussian mixture forms,

(12) $$v_{k-1\left|k\right.}(x)=\sum_{i=1}^{J_{k-1}}w_{k-1\left|k\right.}^{\left(i\right)}N(x;m_{k-1\left|k\right.}^{\left(i\right)},P_{k-1\left|k\right.}^{\left(i\right)})$$

(2) Update to the GM-CPHD filter

The predicted k-time intensity is in a Gaussian mixture form:

(13) $$v_{k+1\left|k\right.}(x)=(1+p_{d,k})v_{k\left|k-1\right.}(x)+\sum_{z\in Z_{k}}v_{d,k}(x;z)$$

Among this set are the following:

(14) $$v_{d,k}(x;z)=\sum_{j=1}^{J_{k\left|k-1\right.}}w_{k}^{j}N(x;m_{k+1\left|k\right.}^{j},P_{k+1\left|k\right.}^{j})$$

(15) $$\omega_{k}^{j}(z)=\frac{p_{d,k}\omega_{k-1\left|k\right.}^{j}q_{k}^{j}(z)}{\kappa_{k}(z)+p_{d,k}\sum_{l=1}^{J_{k-1\left|k\right.}}w_{k-1\left|k\right.}^{l}q_{k}^{l}(z)}$$

(16) $$m_{k+1\left|k\right.}^{j}(z)=m_{k-1\left|k\right.}^{j}+K_{k}^{j}(z-H_{k}m_{k-1\left|k\right.}^{j})$$

(17) $$P_{k+1\left|k\right.}^{j}=[I-K_{k}^{j}H_{k}]P_{k-1\left|k\right.}^{j}$$

(18) $$K_{k}^{j}=P_{k-1\left|k\right.}^{j}H_{k}^{T}{\left(H_{k}P_{k-1\left|k\right.}^{j}H_{k}^{T}+R_{k}\right)}^{-1}$$

where $H_{k}$ is the observation matrix and $R_{k}$ is the measurement noise covariance matrix.

### 2.2. GM-JMNS-CPHD Filter

Based on the research Assumptions 1–3 above, when the JMNS (jump Markov nonlinear system) is applied to CPHD filters, there is no target generation model in the model, and the target generation model and clutter model must include a probability distribution of the number of new targets and the number of clutter measurements. Assumptions 1 and 2 must meet the following requirements [30,31].

#### 2.2.1. JMNS-CPHD Filtering

(1) Prediction of the JMNS-CPHD Filter

(19) $$v_{k-1\left|k\right.}(\ddot{x})=r_{k-1\left|k\right.}(\ddot{x})+\int p_{s}({\ddot{x}}^{\prime })\cdot v_{k-1\left|k\right.}(\ddot{x})\cdot f_{k-1\left|k\right.}(\ddot{x}\left|{\ddot{x}}^{\prime }\right.)d\ddot{x}$$

where $b_{k-1\left|k\right.}(\ddot{x})$ $f_{k-1\left|k\right.}(\ddot{x}\left|{\ddot{x}}^{\prime }\right.)$ represents respectively intensity function during the appearance of the target, probability of target survival, and jumping Markov transition density.

(20) $$p_{k-1\left|k\right.(n)}=\sum_{n^{\prime }\geq 0}\sum_{l=j}^{\infty }C_{j}^{l}p(l)\frac{{\langle p_{s,k},D\rangle }^{j}{\langle 1-p_{s,k},D\rangle }^{j}}{{\langle 1,D\rangle }^{l}}\cdot p_{k\left|k\right.(n^{\prime })}$$

At this point, it can be rewritten as

(21) $$v_{k-1\left|k\right.(x,o)}=r_{k-1\left|k\right.(x,o)}+{v^{\prime }}_{k-1\left|k\right.(x,o)}$$

(22) $${v^{\prime }}_{k-1\left|k\right.(x,o)}=\sum_{o^{\prime }}X_{o,o^{\prime }}\int p_{s}(x^{\prime },o^{\prime })\cdot v_{k-1\left|k\right.}(x^{\prime },o^{\prime })\cdot f_{k-1\left|k\right.}(x^{\prime }\left|x^{\prime },o^{\prime }\right.)dx^{\prime }$$

(2) Update to the JMNS-CPHD filter

The update to the JMNS-CPHD filter can be expressed as follows below.

Given the distribution of cardinality predictions 1 and 2, the update function of the PHD at time k can be expressed as

(23) $$p_{k\left|k\right.}(n)=\frac{r_{k}^{0}\left[v_{k+1\left|k\right.},Z_{k}\right](n)p_{k+1\left|k\right.}(n)}{\langle r_{k}^{0}\left[v_{k+1\left|k\right.},Z_{k}\right],p_{k+1\left|k\right.}\rangle }$$

(24) $$v_{k\left|k\right.}(x,o)=(1-p_{k\left|k\right.}(x,o))\frac{\langle b_{k}^{1}\left[v_{k+1\left|k\right.},Z_{k}\right],p_{k+1\left|k\right.}\rangle }{\langle b_{k}^{0}\left[v_{k+1\left|k\right.},Z_{k}\right],p_{k+1\left|k\right.}\rangle }v_{k+1\left|k\right.}(x,o)+{v^{\prime }}_{k\left|k\right.}(x,o)$$

(25) $${v^{\prime }}_{k\left|k\right.}(x,o)=\sum_{z\in Z_{k}}\frac{\langle r_{k}^{1}\left[v_{k+1\left|k\right.},\frac{Z_{k}}{\left\{z\right\}}\right],p_{k+1\left|k\right.}\rangle }{\langle r_{k}^{0}\left[v_{k+1\left|k\right.},Z_{k}\right],p_{k+1\left|k\right.}\rangle }\frac{\langle 1,\kappa_{k}\rangle g_{k}(z\left|x,o\right.)p_{D,k}(x,o)}{\kappa_{k}(z)}v_{k+1\left|k\right.}(x,o)$$

For which

(26) $$r_{k}^{u}\left[v,Z\right](n)=\sum_{j=0}^{\min(\left|Z\right|,n)}\left(\left|Z\right|-j\right)!p_{k}^{\kappa }(\left|Z\right|-j)P_{j+u}^{n}\times \frac{{\langle 1-p_{v,k},v\rangle }^{n-(j+u)}}{{\langle 1,v\rangle }^{n}}e_{j}\left\{\langle D,\varphi_{k,z}\rangle :z\in Z\right\}$$

where $e_{j}(Z)$ represents an elementary symmetric function:

(27) $$e_{j}(Z)=\sum_{S\subseteq Z,\left|S\right|=j}\prod_{i=S^{i}}ie_{0}(Z)=1$$

where O is integrated as an interference variable to obtain the target’s CPHD separately:

(28) $$v_{k+1\left|k+1\right.}(x)=\sum_{o}v_{k+1\left|k+1\right.}(x,o)$$

#### 2.2.2. Gaussian Mixture JMNS-CPHD Filtering

As early as 2011, relevant scholars conducted research and analysis on GM-JNS-CPHD [32].

(1) Prediction of the GM-JMNS-CPHD Filter

The Gaussian distribution forms of $r_{k+1\left|k\right.(x,o)}$ and $v_{k-1\left|k\right.(x,o)}$ for new targets are

(29) $$b_{k+1\left|k\right.(x,o)}=\sum_{j=1}^{J_{B,o}}b_{k}^{j}(o)N(x;m_{B,k}^{j}(o),P_{B,k}^{j}(o))$$

(30) $$v_{k-1\left|k\right.(x,o)}=\sum_{i=1}^{J_{o,k-1}}{v^{\prime }}_{k-1\left|k\right.}(o)N(x;m_{k-1}^{i}(o),P_{k-1}^{i}(o))$$

$p_{k+1\left|k\right.(n)}$ ${\bar{p}}_{k+1\left|k\right.(n\left|n^{\prime }\right.)}$ can be expressed as

(31) $$p_{k+1\left|k\right.(n)}=\sum_{n^{\prime }\geq 0}p_{k+1\left|k\right.(n\left|n^{\prime }\right.)}\cdot {\bar{p}}_{k\left|k\right.(n^{\prime })}$$

The Markov transition probability ${\bar{p}}_{k+1\left|k\right.(n\left|n^{\prime }\right.)}$ is

(32) $${\bar{p}}_{k+1\left|k\right.(n\left|n^{\prime }\right.)}=\sum_{l=j}^{\infty }C_{j}^{l}p(l)\frac{\sum_{o}p_{s}(o)\sum_{i=1}^{J_{o,k-1}}{v^{\prime }}_{k-1\left|k\right.}^{i}(o)\cdot \Gamma^{j}\langle 1-p_{s,k},v\rangle }{\Gamma^{l}\langle 1,v\rangle }$$

At this point, it can be rewritten as:

(33) $$v_{k+1\left|k\right.(x,o)}=r_{k+1\left|k\right.(x,o)}+\sum_{o^{\prime }}\sum_{i=1}^{J_{o,k-1}}X_{o,o^{\prime }}p_{s}(x^{\prime },o^{\prime }){v^{\prime }}_{k-1}^{i}(x^{\prime },o^{\prime })N(x;M^{\prime })$$

Among them:

(34) $$M^{\prime }=\frac{\sum_{j=1}^{M}x_{+}^{i,j}}{M},\frac{\sum_{j=1}^{M}\left[m_{S,+}^{i}(o^{\prime })-x_{+}^{i,j}\right]{\left[m_{S,+}^{i}(o^{\prime })-x_{+}^{i,j}\right]}^{T}}{M}$$

(35) $$x_{+}^{i,j}∼f_{+}(x\left|x_{k-1}^{i,j},o^{\prime }\right.)x_{k-1}^{i,j}∼N(x;m_{k-1}^{i}(o^{\prime }),p_{k-1}^{i}(o^{\prime }))$$

(2) Update to the GM-JMNS-CPHD filter

The update to the GM-JMNS-CPHD filter can be expressed as follows below [33,34].

Given the distribution of the cardinality predictions $p_{k+1\left|k\right.(n)}$ and $v_{k+1\left|k\right.(x,o)}$, the update function of the PHD at time k can be expressed as

(36) $$p_{k\left|k\right.}(n)=\frac{b_{k}^{0}\left[v_{k+1\left|k\right.},Z_{k}\right](n)p_{k+1\left|k\right.}(n)}{\langle b_{k}^{0}\left[v_{k+1\left|k\right.},Z_{k}\right],p_{k+1\left|k\right.}\rangle }$$

(37) $$v_{k\left|k\right.}(x,o)=\sum_{z\in Z_{k}}\sum_{i=1}^{J_{0,+}}\alpha_{k}^{i}(z,o)\beta_{z}(o)\delta_{z}^{i}(x,o)+(1-p_{k\left|k\right.}(x,o))v_{k+1\left|k\right.}(x,o)\frac{\langle r_{k}^{1}\left[v_{k+1\left|k\right.},Z_{k}\right],p_{k+1\left|k\right.}\rangle }{\langle r_{k}^{0}\left[v_{k+1\left|k\right.},Z_{k}\right],p_{k+1\left|k\right.}\rangle }$$

(38) $$\alpha_{k}^{i}(z,o)=\frac{1}{M^{\prime }}\sum_{j=1}^{M^{\prime }}\frac{u_{k}(z\left|x_{k}^{i,j},o\right.)N(x_{k}^{i,j};m_{+}^{i}(o),P_{+}^{i}(o))}{\pi_{k}^{i}(x_{k}^{i,j}\left|Z_{1:k-1},z,o\right.)}$$

(39) $$\beta_{z}(o)=p_{k\left|k\right.}(x,o)\frac{\langle 1,\kappa_{k}\rangle \langle b_{k}^{1}\left[v_{k+1\left|k\right.},Z_{k}\right],p_{k+1\left|k\right.}\rangle }{\kappa_{k}(z)\langle b_{k}^{0}\left[v_{k+1\left|k\right.},Z_{k}\right],p_{k+1\left|k\right.}\rangle }$$

(40) $$\delta_{z}^{i}(x,o)=v_{k+1\left|k\right.}^{i}(o)N(x;m_{k}^{i}(z,o),P_{k}^{i}(z,o)$$

(41) $$P_{K}^{i}(z,o)=\frac{\sum_{j=1}^{M^{\prime }}\frac{g_{k}(z\left|x_{k}^{i,j},o\right.)N(x_{k}^{i,j};m_{+}^{i}(o),P_{+}^{i}(o))}{\pi_{k}^{i}(x_{k}^{i,j}\left|Z_{1:k-1}\right.,z,o)}[m_{k}^{i}(z,o)-x_{k}^{i,j}]{\left[m_{k}^{i}(z,o)-x_{k}^{i,j}\right]}^{T}}{\sum_{j=1}^{M^{\prime }}\frac{g_{k}(z\left|x_{k}^{i,j},o\right.)N(x_{k}^{i,j};m_{+}^{i}(o),P_{+}^{i}(o))}{\pi_{k}^{i}(x_{k}^{i,j}\left|Z_{1:k-1}\right.,z,o)}}$$

At this point, $x_{k}^{i,j}∼\pi_{k}^{i}(\cdot \left|Z_{1:k-1}\right.,z,o)$, which is represented as a probability distribution of $x_{k}^{i,j}$, obeys $\pi_{k}^{i}(\cdot \left|Z_{1:k-1}\right.,z,o)$.

### 2.3. Integration Criteria

The commonly used ellipsoidal methods for multisensor fusion include CI, ICI, BC, LE, and other methods. To verify the effectiveness of our algorithm, multiple ellipsoidal methods for multisensor fusion were compared. The CI and ICI fusion methods demonstrated good results. Therefore, this study investigated two fusion strategies, the CI and ICI.

#### 2.3.1. CI Fusion Strategy

Covariance Intersection (CI) [35,36] fusion: in two sensor systems, if the subsystem estimation error variance matrix $P_{1},P_{2}$ is known and the cross covariance $P_{1,CI},P_{2,CI}$ is unknown, the covariance cross-fusion algorithm is

(42) $$x_{CI}=P_{CΙ}(\omega_{CI}P_{1}^{-1}x_{1}+(1-\omega_{CI})P_{2}^{-1}x_{2})=P_{CI}(P_{1,CI}^{-1}x_{1}+P_{2,CI}^{-1}x_{2})$$

(43) $$P_{CI}=(\omega_{CI}P_{1}^{-1}+(1-\omega_{CI})P_{2}^{-1}{)}^{-1}=(P_{1,CI}^{-1}+P_{2,CI}^{-1}{)}^{-1}$$

(44) $$P_{1,CI}^{-1}≜\omega_{CI}P_{1}^{-1}$$

(45) $$P_{2,CI}^{-1}≜(1-\omega_{CI})P_{2}^{-1}$$

As such,

(46) $$\min J=\underset{\omega \in [0,1]}{\min}trP_{CI}=\underset{\omega \in [0,1]}{\min}tr\left\{{\left[\omega {\left(P_{1}\right)}^{-1}+(1-\omega ){\left(P_{2}\right)}^{-1}\right]}^{-1}\right\}$$

#### 2.3.2. ICI Fusion Strategy

Inverse covariance intersection (ICI) [37] fusion: in two sensor systems, if the subsystem estimation error variance matrix is known and the cross covariance is unknown, the inverse covariance cross-fusion algorithm is

(47) $$x_{ICI}=K_{ICΙ}x_{1}+L_{ICΙ}x_{2}$$

(48) $$P_{ICI}=P_{1,ICI}^{-1}+P_{2,ICI}^{-1}-{\left(\omega_{ICI}P_{1}+(1-\omega_{ICI})P_{2}\right)}^{-1}$$

(49) $$K_{ICI}=P_{ICI(}P_{1}^{-1}-\omega_{ICI}{\left(\omega_{ICI}P_{1}+(1-\omega_{ICI})P_{2}\right)}^{-1}$$

(50) $$L_{ICI}=P_{ICI(}P_{2}^{-1}-(1-\omega_{ICI}){\left(\omega_{ICI}P_{1}+(1-\omega_{ICI})P_{2}\right)}^{-1}$$

As such,

(51) $$\begin{array}{l} \min J=\underset{\omega \in [0,1]}{\min}trP_{ICI}\\ =\underset{\omega \in [0,1]}{\min}tr\left\{{\left[{\left(P_{1}\right)}^{-1}+{\left(P_{2}\right)}^{-1}-(\omega P_{1}+(1-\omega ){\left(P_{2}\right)}^{-1}\right]}^{-1}\right\} \end{array}$$

## 3. Application of a Generalized Covariance Intersection for Multitarget Tracking in the GM-JMNS-CPHD

### 3.1. GCI-GM-JMNS-CPHD

Two Gaussian components, $x∼N({\hat{x}}_{1},{\hat{P}}_{1})$ and $x∼N({\hat{x}}_{2},{\hat{P}}_{2})$, using the CI strategy, can be expressed as

(52) $$N_{{\hat{p}}_{\omega }}(x-{\hat{x}}_{\omega })=\frac{{\left[N_{{\hat{p}}_{1}}(x-{\hat{x}}_{1})\right]}^{\omega }{\left[N_{{\hat{p}}_{2}}(x-{\hat{x}}_{2})\right]}^{1-\omega }}{\int {\left[N_{{\hat{p}}_{1}}(x-{\hat{x}}_{1})\right]}^{\omega }{\left[N_{{\hat{p}}_{2}}(x-{\hat{x}}_{2})\right]}^{1-\omega }dx}$$

where ω∈[0,1].

The GCI fusion strategy is a generalized rule that combines multiobjective density functions with arbitrary densities. The GCI strategy can be described as follows:

(53) $$f_{\omega }(x\left|G_{1}^{k}\right.,G_{2}^{k})≜\frac{{\left[f_{1}(x\left|G_{1}^{k}\right.)\right]}^{\omega }{\left[{\left[f_{2}(x\left|G_{2}^{k}\right.)\right]}^{\omega }\right]}^{1-\omega }}{\int {\left[f_{1}(x\left|G_{1}^{k}\right.)\right]}^{\omega }{\left[{\left[f_{2}(x\left|G_{2}^{k}\right.)\right]}^{\omega }\right]}^{1-\omega }dx}$$

Assuming that s(x) is a local density function, the local multiobjective density that should be fused into the GM-JMNS-CPHD is

(54) $$f_{1}(X)=n!p_{1}(n)\prod_{x\notin X}s_{1}(x)$$

(55) $$f_{2}(X)=n!p_{2}(n)\prod_{x\notin X}s_{2}(x)$$

The GCI fusion strategy can be applied to the GM-JMNS-CPHD as follows:

(56) $$s(x)=\frac{s_{1}^{\omega }(x)s_{2}^{1-\omega }(x)}{\int s_{1}^{\omega }(x)s_{2}^{1-\omega }(x)dx}$$

(57) $$p(n)=\frac{p_{1}^{\omega }(n)p_{2}^{1-\omega }(n){\left(s_{1}^{\omega }(x)s_{2}^{1-\omega }(x)\right)}^{n}}{\sum_{m=0}^{\infty }p_{1}^{\omega }(m)p_{2}^{1-\omega }(m){\left(\int s_{1}^{\omega }(x)s_{2}^{1-\omega }(x)dx\right)}^{m}}$$

The GM-GCI integration strategy can be described as follows:

(58) $$s_{GCI}(x)=\frac{s_{1}^{\omega }(x)s_{2}^{1-\omega }(x)}{\int s_{1}^{\omega }(x)s_{2}^{1-\omega }(x)dx}=\frac{\sum_{i=1}^{N_{G}^{1}}\sum_{j=1}^{N_{G}^{2}}\alpha_{ij}^{12}N({\hat{x}}_{ij}^{12},P_{ij}^{12})}{\sum_{i=1}^{N_{G}^{1}}\sum_{j=1}^{N_{G}^{2}}\alpha_{ij}^{12}}$$

(59) $$P_{ij}^{12}={\left[\omega_{GCI}{\left(P_{i}^{1}\right)}^{-1}+(1-\omega_{GCI}){\left(P_{j}^{2}\right)}^{-1}\right]}^{-1}$$

(60) $${\hat{x}}_{ij}^{12}=P_{ij}^{12}[\omega_{GCI}{\left(P_{i}^{1}\right)}^{-1}{\hat{x}}_{i}^{1}+(1-\omega_{GCI}){\left(P_{j}^{2}\right)}^{-1}{\hat{x}}_{j}^{2}]$$

(61) $$\alpha_{ij}^{12}={\left(\alpha_{i}^{1}\right)}^{\omega }{\left(\alpha_{j}^{2}\right)}^{1-\omega }\kappa (\omega ,P_{i}^{1})\kappa (1-\omega ,P_{j}^{2})N({\hat{x}}_{i}^{1}-{\hat{x}}_{j}^{2};0;\frac{P_{i}^{1}}{\omega }+\frac{P_{j}^{2}}{1-\omega })$$

The GCI-GM-JMNS-CPHD algorithm process is shown in Algorithm 1. The GM-JMNS-CPHD is calculated according to Formulas (25)–(38) above, and its weight is calculated according to the GCI fusion algorithm. Finally, the fusion result is modified and improved.
**Algorithm 1:** GCI-GM-JMNSCPHD filtering algorithm process.1. Calculate the distribution GM-JMNS-CPHD results according to Formulas (25)–(38), calculate the prediction of GM-JMNS-CPHD, and update 2. For M sensors 3. Using the Formulas (56)–(58) GCI fusion strategy to calculate weights $\omega_{GCI}$
4. Calculate different $P_{k\left|k\right.}^{1(m)},P_{k\left|k\right.}^{2(m)}$ separately 5. Calculate the next level fusion result based on the previous level fusion result 6. Modify and improve GCI-GM-CPHD through “pruning” and “merging” 7. end for 8. Estimate extraction

### 3.2. GICI-GM-JMNS-CPHD

WOO JUNG PARK [38] (2021) proposed a generalized inverse covariance dilation method, and this study used this method for its calculations. The core of the inflation method is the removal of the weight part of the CI fusion strategy and the change of the original structure of the ICI to

(62) $$x_{ICI}=K_{ICΙ}x_{1}+L_{ICΙ}x_{2}$$

(63) $$P_{ICI}={\left(P_{1,ICI}^{-1}+P_{2,ICI}^{-1}\right)}^{-1}$$

(64) $$P_{1,ICI}^{-1}≜P_{1}^{-1}-\omega_{ICI}{\left(\omega_{ICI}P_{1}+(1-\omega_{ICI})P_{2}\right)}^{-1}$$

(65) $$P_{2,ICI}^{-1}≜P_{2}^{-1}-(1-\omega_{ICI}){\left(\omega_{ICI}P_{1}+(1-\omega_{ICI})P_{2}\right)}^{-1}$$

The verification of the above scholars demonstrates that $x^{T}P_{1,CI}x>0,\;x^{T}P_{2,CI}x>0$ and $P_{1,CI},P_{2,CI}$ have positive definiteness.

(66) $$x^{T}P_{1,ICI}x=x^{T}P_{1}x+\frac{\omega }{1-\omega }y^{T}P_{2}^{-1}y>0$$

(67) $$x^{T}P_{2,ICI}x=x^{T}P_{B}x+\frac{\omega }{1-\omega }y^{T}P_{A}^{-1}y>0$$

The GM-GICI fusion strategy can be described as follows:

(68) $${\hat{s}}_{GCI}(x)=\frac{s_{1}(x)s_{2}(x)}{\int s_{1}(x)s_{2}(x)dx}=\frac{\sum_{i=1}^{N_{G}^{1}}\sum_{j=1}^{N_{G}^{2}}\alpha_{ij}^{12}N({\hat{x}}_{ij}^{12},P_{ij}^{12})}{\sum_{i=1}^{N_{G}^{1}}\sum_{j=1}^{N_{G}^{2}}\alpha_{ij}^{12}}$$

(69) $$P_{ij}^{12}={\left[{\left(P_{i}^{1}\right)}^{-1}+{\left(P_{j}^{2}\right)}^{-1}\right]}^{-1}$$

(70) $${\hat{x}}_{ij}^{12}=P_{ij}^{12}[{\left(P_{i}^{1}\right)}^{-1}{\hat{x}}_{i}^{1}+({\left(P_{j}^{2}\right)}^{-1}{\hat{x}}_{j}^{2}]$$

(71) $$\alpha_{ij}^{12}={\left(\alpha_{i}^{1}\right)}^{\omega }{\left(\alpha_{j}^{2}\right)}^{1-\omega }N({\hat{x}}_{i}^{1}-{\hat{x}}_{j}^{2};0;P_{i}^{1}+P_{j}^{2})$$

The GICI-GM-JMNSCPHD algorithm process is shown in Algorithm 2. The GM-JMNSCPHD is also calculated according to Formulas (25)–(38) above, and its weight is calculated according to the GICI fusion algorithm. Finally, the fusion result is modified and improved.
**Algorithm 2:** GICI-GM-JMNSCPHD filtering algorithm process.1. Calculate the distribution GM-JMNSCPHD results according to Formulas (28)–(41), calculate the prediction of GM-JMNSCPHD, and update 2. For M sensors 3. Using the Formulas (59)–(61) GCI fusion strategy to calculate weights $\omega_{GICI}$
4. Replace the covariance of a single sensor probability density of $P_{1}^{y},P_{2}^{y}$ with $\left\{\begin{array}{l} P_{1,ICI}^{m}=P_{1}^{m}+\frac{\omega_{ICI}}{1\;-\;\omega_{ICI}}P_{1}^{m}{\left(P_{2}^{m}\right)}^{-1}P_{A}^{m}\\ P_{2,ICI}^{m}=P_{2}^{m}+\frac{\omega_{ICI}}{1\;-\;\omega_{ICI}}P_{B}^{m}{\left(P_{A}^{m}\right)}^{-1}P_{B}^{m} \end{array}\right.$ 5. Calculate the next level fusion result based on the previous level fusion result 6. Calculate GM covariance through (54)–(61) 7. Modify and improve GCI-GM-CPHD through “pruning” and “merging” 8. End for 9. Estimate extraction

## 4. Modeling and Simulation

This study adopts the GM-JMNS-CPHD filter as its research focus and mainly explores the fusion strategies of nonlinear Gaussian models. For this purpose, nonlinear models are selected and simulated using the constant turn rate and velocity (CTRV) model. The nonlinear CTRV model is $CTRV(x)={\left(x\;\vartheta \;y\;\psi \;\dot{\psi }\right)}^{⊤}$, where the five variables are as follows: x is the abscissa, y is the ordinate, v is the line velocity, ψ is the yaw angle (counterclockwise to the included angle), and $\dot{\psi }$ is the angular velocity.

(72) $$x_{k}=\left[\begin{array}{ccccc} 1 & \frac{\sin\Omega T}{\Omega } & 0 & -\frac{1\;-\;\cos\Omega T}{\Omega } & 0\\ 0 & \cos\Omega T & 0 & -\sin\Omega T & 0\\ 0 & \frac{1\;-\;\cos\Omega T}{\Omega } & 1 & \frac{\sin\Omega T}{\Omega } & 0\\ 0 & \sin\Omega T & 0 & \cos\Omega T & 0\\ 0 & 0 & 0 & 0 & 1 \end{array}\right]x_{k-1}+v_{k}$$

The measurement noise is

(73) $$wk∼N(0,R),\;R=diag[\sigma_{r}^{2}\sigma_{\theta }^{2}]$$

(74) $$Q=diag[q_{1}M\;q_{1}M\;q_{1}T]$$

To verify the effectiveness of this study, the generalized covariance intersection of multiobjective tracking is applied to several aspects of the GM-JMNS-CPHD algorithm.

### 4.1. Effectiveness of the GM-JMNS-CPHD Algorithm

The GM-JMNS-CPHD filter is applied to a sensor with a detection probability of 0.9 and a survival probability of 0.99. The Poisson average rate of the uniform clutter of the moving target is 8, and its birth density is located at (±2000 m, ±2000 m). All simulations are completed using 200 Monte Carlo experiments, with the truncation threshold $T=10^{-5}$, merging threshold U=2, and maximum allowable number of Gaussian terms $J_{\max}=100$ set in the GM-JMNS-CPHD algorithm. The tracking performance of the moving targets is measured by their optimal subpattern allocation (OSPA) distance. The parameters for the OSPA distance are set as a cutoff parameter c=100 and order parameter p=1. A nonlinear Gaussian measurement model is selected, and the initial state of the target in the nonlinear linear Gaussian measurement model is shown in Table 1.

**Figure 1:**
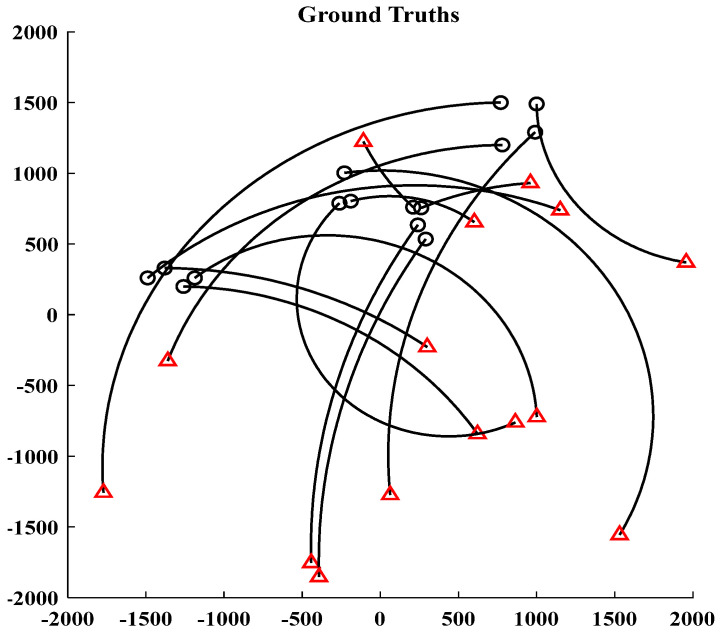
Nonlinear CTRV model of a moving target’s trajectory. The circle represents the target time of birth, and the triangle represents the time of death.

**Figure 2:**
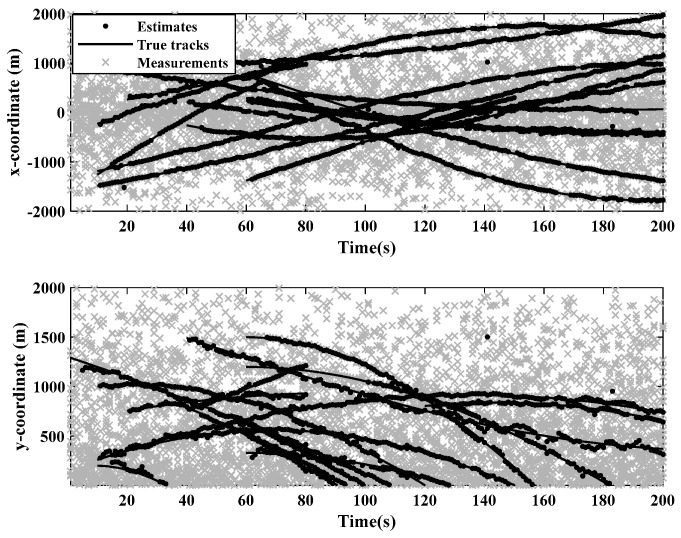
True track measurement values and the estimated values of the target’s Cartesian coordinates in nonlinear models.

**Figure 3:**
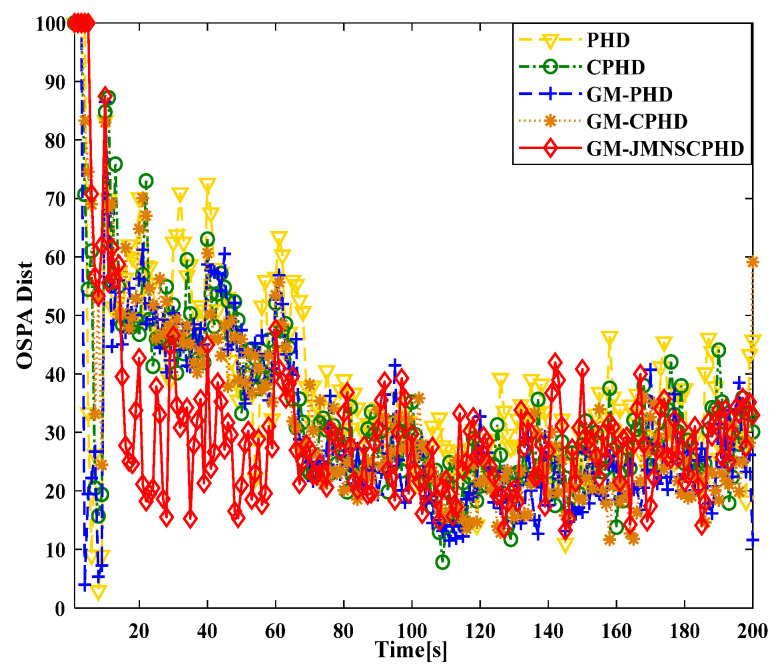
Comparison of OSPA distance errors between several algorithms used in nonlinear models.

**Figure 4:**
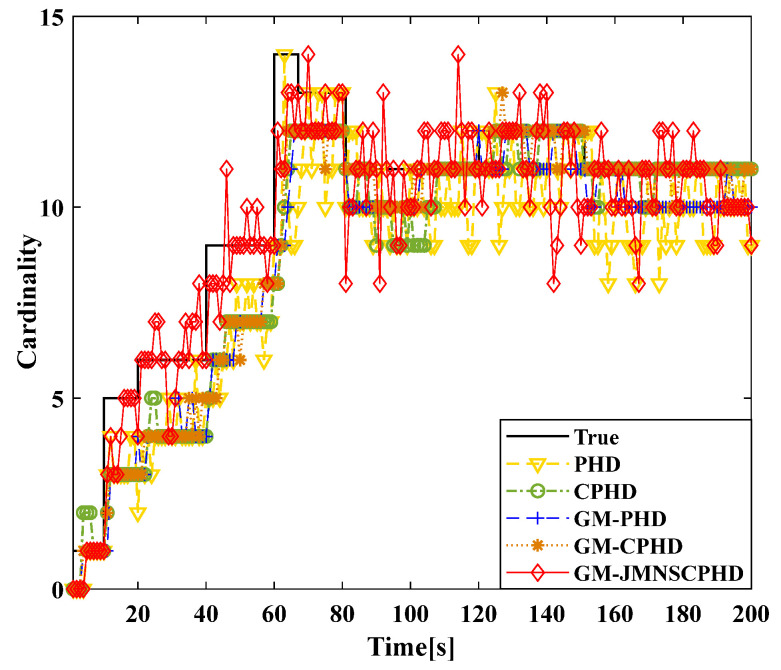
Comparison of the cardinal distributions of several algorithms used in nonlinear models.

Figure 1 and Figure 2 show the moving target’s trajectory in the nonlinear CTRV model, as well as its true trajectory, measured values, and estimated values in the Cartesian coordinate system of the nonlinear model. The GM-JMNS-CPHD has good performance in the multitarget tracking of nonlinear systems and is suitable for the multitarget tracking of nonlinear systems. From a comparison of the OSPA distance errors between the algorithms in the nonlinear model shown in Figure 3 and a comparison of the cardinality distributions between the algorithms in the nonlinear model shown in Figure 4, it can be seen that the GM-JMNS-CPHD has a good implementation effect and can be used for multiobjective nonlinear motion tracking.

**Figure 5:**
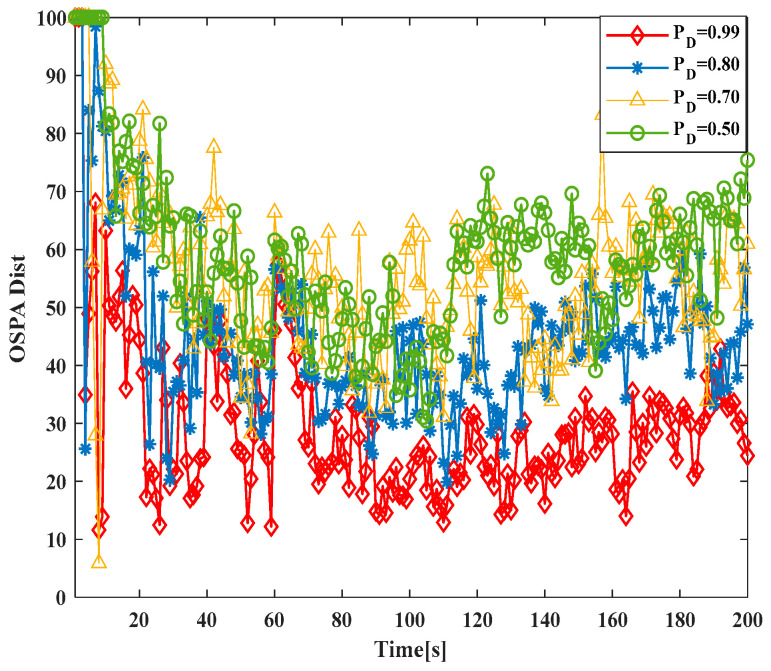
Comparison of OSPA distance errors due to the impact of different object detection probabilities on algorithm performance.

**Figure 6:**
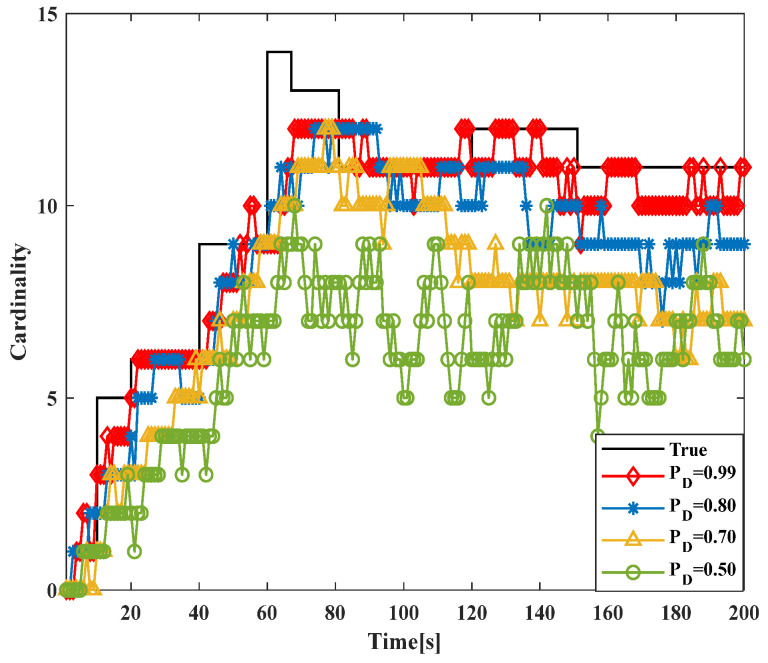
Comparison of the cardinal distribution of different object detection probabilities on algorithm performance.

To verify the robustness of the GM-JMNS-CPHD, this study will conduct research and analysis on the performance of the algorithm under different noise levels. The impact of different object detection probabilities $p_{D}$ on the algorithm’s performance will be tested in different scenarios, and the effects under different conditions of $p_{D}$ will be determined, as shown in Figure 5 and Figure 6. The results in the figure show that the GM-JMNS-CPHD can maintain its stability at the same noise level and that it has a certain degree of robustness.

### 4.2. Implementation of the GICI-GM-JMNS-CPHD Algorithm

**Figure 7:**
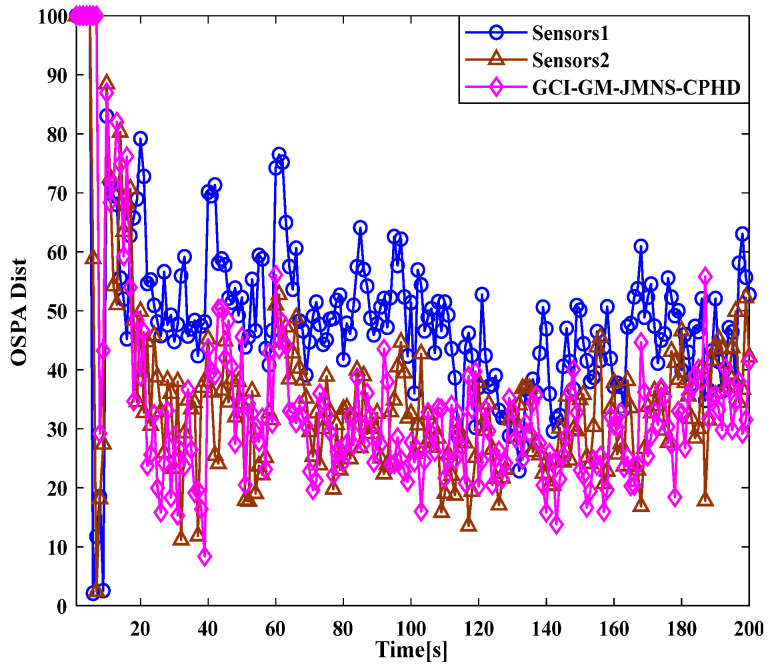
Comparison of OSPA distance errors in nonlinear models using GCI-GM-JMNS-CPHD.

**Figure 8:**
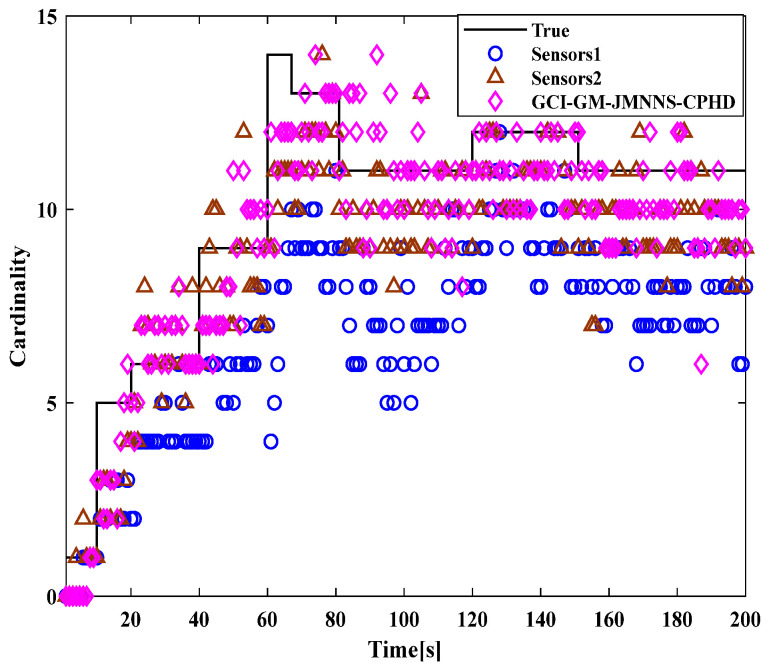
Comparison of the cardinal distributions of nonlinear models using GCI-GM-JMNS-CPHD.

After verifying the effectiveness of the GM-JMNS-CPHD filter, the GCI and GICI fusion strategies were applied to the GM-JMNS-CPHD filter. Because the fusion performance of the GCI and GICI sensors is proportional to the number of sensors, this study selected two sensors as the research objects to verify the target tracking performance of the Sensor1, Sensor2, GCI-GM-JMNS-CPHD, and GCI-GM-JMNS-CPHD methods. According to the above GCI-GM-JMNSCPHD filtering algorithm flow and GCI-GM-JMNSCPHD filtering algorithm flow, the algorithm flow is implemented, as shown in Figure 7 and Figure 8.

A comparison of the OSPA distance errors in the nonlinear model in Figure 7, using the GCI-GM-JMNS-CPHD, and a comparison of the cardinality distributions in the nonlinear model in Figure 8, using the GCI-GM-JMNS-CPHD, reveals that the GCI fusion criterion can be applied to the GM-JMNS-CPHD algorithm. Compared with that of the GCI-GM-JMNS-CPHD, the performances of the single sensors Sensor1 and Sensor2 in nonlinear multitarget tracking can be improved.

**Figure 9:**
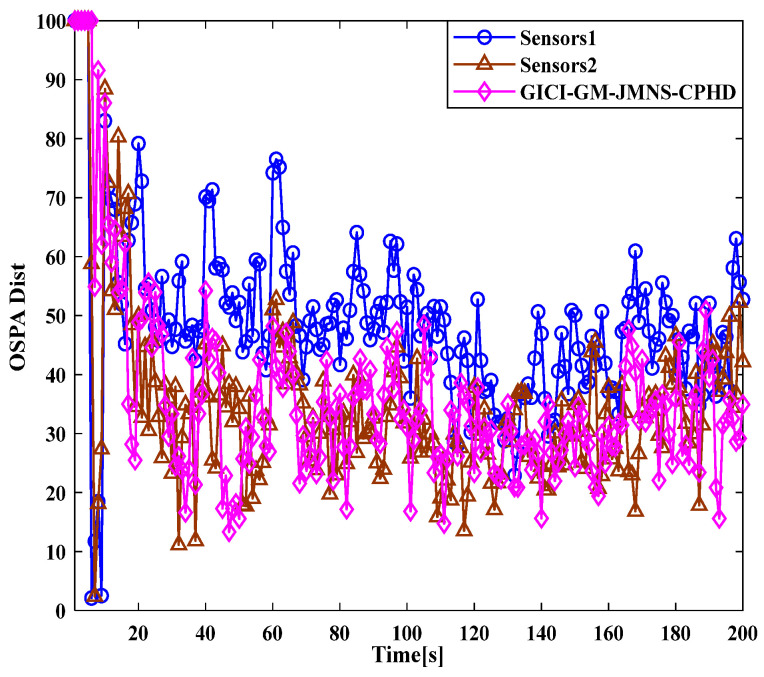
Comparison of OSPA distance errors between several algorithms used in nonlinear models.

**Figure 10:**
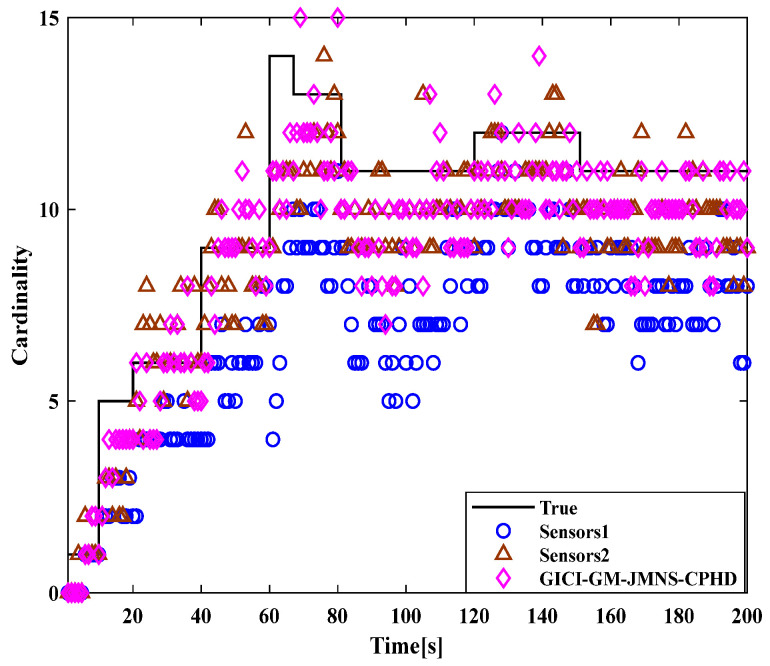
Comparison of the cardinal distributions of several algorithms used in nonlinear models.

By comparing the OSPA distance errors in the nonlinear model in Figure 9 and the cardinality distributions in the nonlinear model in Figure 10, it can be seen that the GICI fusion criterion can be applied to the GM-JMNS-CPHD algorithm. Compared to the GICI-GM-JMNS-CPHD, the proposed sensor can improve the performance of nonlinear multitarget tracking in comparison to the single-sensor performances of Sensor1 and Sensor2.

**Figure 11:**
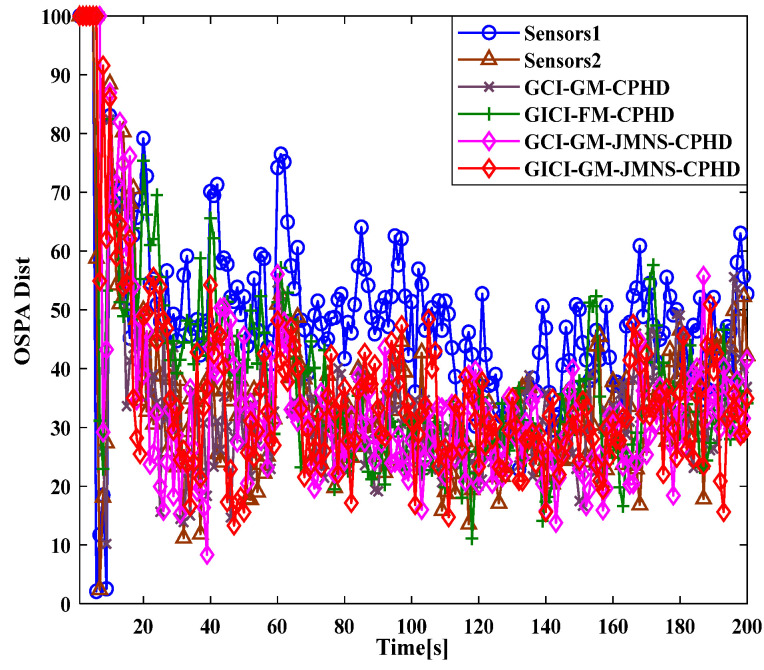
Comparison of OSPA distance errors between several GM-CPHD algorithms commonly used in nonlinear models.

**Figure 12:**
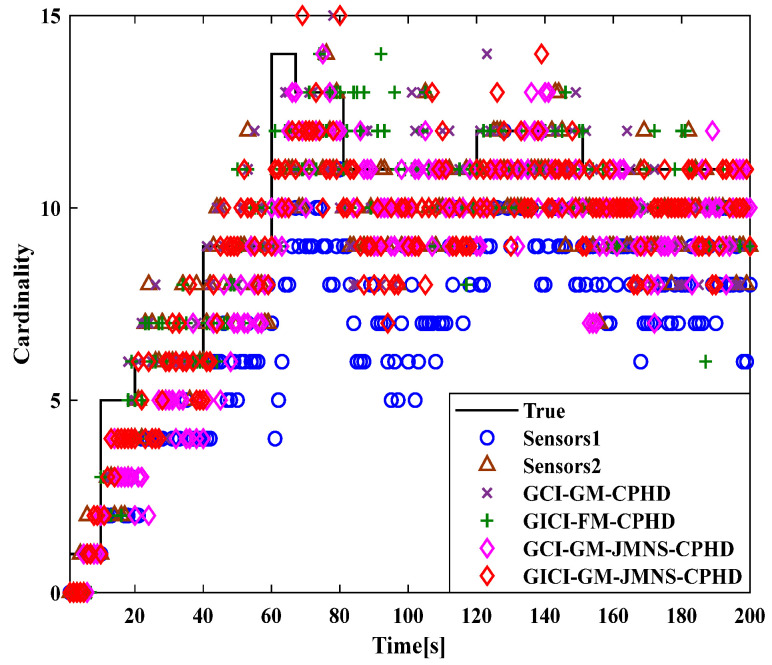
Comparison of the cardinal distributions of several commonly used nonlinear GM-CPHD algorithms.

To compare the effectiveness of the GCI-GM-JMNS-CPHD and GICI-GM-JMNS-CPHD methods, this study selected the Sensor1, Sensor2, GCI-GM CPHD, GICI-GM-CPHD, GCI-GM-JMNS-CPHD, and GICI-GM-JMNS-CPHD methods for comparison, as is shown in Figure 11 and Figure 12.

From the comparison of the OSPA distance errors of several commonly used GM-CPHD algorithms in nonlinear models, seen in Figure 11 and Figure 12, and the comparison of the cardinality distributions of several commonly used GM-CPHD algorithms in nonlinear models, seen in Figure 11 and Figure 12, it can be seen that, compared to those of other methods such as the GCI-GM-CPHD and GICI-GM-CPHD, the errors of these algorithms are relatively large, while the direct effects of these two methods on nonlinear multimotion target tracking are not significant. The comparison results between the GCI-GM-JMNS-CPHD and GICI-GM-JMNS-CPHD are relatively similar, and their fused results can outperform the multitarget tracking performance of the Sensor1 and Sensor2 single sensors.

## 5. Summary and Prospects

This study proposes a fusion method based on the GICI-GM-JMNS-CPHD filter, which applies the GCI and GICI fusion criteria to the GM-JMNS-CPHD filter to achieve the multitarget tracking of the GM-CPHD in multisensor and multitarget nonlinear motion. In the simulation experiment, the effectiveness of the two methods was first verified. Second, the comparison between the GCI-GM-JMNS-CPHD and GICI-GM-JMNS-CPHD shows that both have a better performance than the single-sensor multitarget tracking of Sensor1 and Sensor2. The performances of the CI-GM-JMNS-CPHD and GICI-GM-JMNS-CPHD in nonlinear multitarget tracking are superior to those of the traditional fusion methods of the GCI-GM-CPHD and GICI-GM-CPHD. This approach is more suitable for the nonlinear motion tracking of multiple moving targets with sensors.

Although the above methods have validated the effectiveness of this study, it has been confirmed that the GCI-GM-JMNS-CPPHD and GICI-GM-JMNS-CPPHD can be applied to multitarget tracking in nonlinear systems. However, it is worth noting that both methods take a significant amount of time. The above methods model noise errors, not sensor data, which may affect the accuracy of the fusion method to a certain extent. Additionally, issues such as sensor bias and suboptimal CIs can also affect fusion accuracy. The limitations and future research directions of this study include studying how to efficiently implement these algorithms and reduce their runtime, as well as considering the impact of other related factors on fusion accuracy.

## Figures and Tables

**Table 1:** Initial state of the target in the nonlinear Gaussian measurement model.

Target	Initial State	Appearing Frame	Disappearing Frame
1	[1000; −10; 1300; −10; wturn/8]	1	truth.K + 1
2	[−1500; 11; 250; 10; −wturn/6]	10	truth.K + 1
3	[−250; 20; 1000; 3; −wturn/3]	10	truth.K + 1
4	[−1200; 12; 250; 10; −wturn/3]	10	truth.K + 1
5	[−1300; 40; 200; 0; −wturn/2]	10	66
6	[250; 11; 750; 5; −wturn/6]	20	80
7	[−250; −12; 800; −12; wturn/2]	40	truth.K + 1
8	[1000; 0; 1500; −10; wturn/4]	40	truth.K + 1
9	[220; −10; 750; 10; −wturn/4]	40	80
10	[800; −20; 1200; 0; wturn/4]	60	truth.K + 1
11	[250; −10; 650; −15; wturn/8]	60	truth.K + 1
12	[−1400; 20; 330; 0; −wturn/5]	60	150
13	[800; −30; 1500; 0; wturn/3]	60	truth.K + 1
14	[300; −10; 550; −15; wturn/8]	80	truth.K + 1
15	[−200; 10; 800; 3; −wturn/3]	120	200

## Data Availability

Data are contained within the article.
